# Recommended nomenclature convention for the NISTCHO cell line and its product monoclonal antibody, cNISTmAb

**DOI:** 10.1080/19420862.2025.2490789

**Published:** 2025-04-11

**Authors:** Megan H. Cleveland, Ioannis L. Karageorgos, John P. Marino, Michael J. Tarlov, Katharina S. Yandrofski, Rebecca A. Zangmeister, Zvi Kelman

**Affiliations:** a Biomolecular Measurement Division, National Institute of Standards and Technology (NIST), Gaithersburg, MD, USA; b Institute for Bioscience and Biotechnology Research (IBBR), University of Maryland, Rockville, MD, USA

**Keywords:** NISTCHO, cNISTmAb, chinese hamster ovary (CHO), reference material, NIST RM, naming convention

## Abstract

NISTCHO is a Chinese hamster ovary (CHO) cell line expressing the same amino acid sequences as the heavy and light chains of the National Institute of Standards and Technology (NIST) monoclonal antibody [Reference Material (RM) 8671 NISTmAb]. NISTCHO was generated by MilliporeSigma to be developed by NIST as a RM to support biomanufacturing research and innovation, method development and qualification, and pre-competitive research collaboration. The RM cell line, denoted as RM 8675 NISTCHO, Clonal CHO-K1 Cell Line Producing cNISTmAb, is of interest to the biopharmaceutical and biomanufacturing industries, regulatory and government agencies, and academic institutions. In contrast to other NIST RMs, however, which are typically discrete and finite, the NISTCHO is a living RM that can be propagated, expanded, and used repeatedly to express the non-originator NISTmAb product, cNISTmAb. Therefore, a uniform naming convention should be adopted by the user community to best track the origins of materials (the cell line and products) used in studies derived from the RM 8675 NISTCHO. Here, we provide a naming convention for the derivatives of the RM 8675 NISTCHO and the cNISTmAb produced by these NISTCHO derivatives and recommend these naming conventions for adoption by the scientific community.

## Introduction

At the Advanced Mammalian Biomanufacturing Innovation Center launch meeting in 2016, industry, government, and academic scientists in attendance concluded that research collaboration and innovation in biomanufacturing was stymied by a lack of an open access, industry-relevant, monoclonal antibody-producing Chinese hamster ovary (CHO) cell line. While CHO cells are commonly used within the biopharmaceutical industry, cell lines developed by companies are typically proprietary and unavailable to the broader scientific community. Furthermore, it was suggested that the National Institute of Standards and Technology (NIST) serve as the host for such a CHO cell line that could be developed into a reference material (RM) for the community.

In 2018, MilliporeSigma agreed to generate such a cell line for NIST.^1^ The cell line, referred to as NISTCHO, was designed to express the same amino acid sequences as the RM 8671 NISTmAb, a humanized IgG1k monoclonal antibody expressed in murine suspension culture. NISTCHO was generated via MilliporeSigma’s CHO-K1 platform (CHOZN GS^−/−^) and therefore produces a non-originator version of the NISTmAb.^2,3^ MilliporeSigma and the National Institute for Innovation in Manufacturing Biopharmaceuticals (NIIMBL) then collaborated with NIST to develop NISTCHO into an industry-relevant, open-access cell line with limited constraints that anyone could purchase on a cost recovery basis from NIST. NIST contracted the production of master (MCB) and working (WCB) cell banks for the NISTCHO to Eurofins Lancaster Laboratories to be produced under Good Manufacturing Practices (GMP) conditions that would be certified as RM 8675 NISTCHO, Clonal CHO-K1 Cell Line Producing cNISTmAb, hereafter referred to as RM 8675 NISTCHO.

In addition to the development of RM 8675 NISTCHO, NIST is also developing a RM based on the product produced by NISTCHO, a CHO-expressed IgG1k monoclonal antibody (cNISTmAb), hereafter referred to as RM 8672 cNISTmAb. NIST contracted with MilliporeSigma to produce cNISTmAb that NIST could then certify as RM 8672 cNISTmAb. The RM 8672 cNISTmAb is a companion to RM 8671 NISTmAb. Because cNISTmAb is made in CHO cells, it has distinguishing post-translational modifications (PTMs), such as glycosylation, as compared to the original RM 8671 NISTmAb which was produced in NS0 murine cells.^3^ Customers may purchase RM 8671 NISTmAb, RM 8672 cNISTmAb, and RM 8675 NISTCHO through the NIST Office of Reference Materials (shop.nist.gov). Taken together, these three companion RMs provide end-to-end support for monoclonal antibody biomanufacturing and characterization to the biopharmaceutical industry, federal laboratories, and academic institutions.

## Why is a naming convention needed?

When using a typical RM, the customer receives a sample of the RM, and when it is depleted, a new sample of the RM must be purchased. Thus, the name provided with the RM, e.g., RM 8671 NISTmAb, can be used to refer to it. In the case of a living reference material such as the RM 8675 NISTCHO, however, this approach is not applicable. When a customer receives the cells, they are encouraged to make their own WCB for future use, which may result in several lineages of propagated cells that are derived from RM 8675 NISTCHO but are no longer the original NISTCHO RM. Therefore, a naming convention that the community can adopt is needed.

Similarly, while NIST is developing RM 8672 cNISTmAb, i.e., the product of RM 8675 NISTCHO, every laboratory that receives RM 8675 NISTCHO will be able to make a non-originator version of NISTmAb. These products will not be the RM 8672 cNISTmAb that is certified and sold by NIST, and thus, as is the case with the cell line RM 8675 NISTCHO, a distinguishing naming convention is needed for the monoclonal antibodies produced.

To this end, we recommend a naming convention for NISTCHO and cNISTmAb that follows several guidelines and assumptions:

- The naming convention should be simple and intuitive so it will be straightforward to follow and adopt.
- Use of the proposed naming convention is most important for outfacing purposes, such as publications, technical notes, and presentations. An individual laboratory may use different naming systems internally.
- The naming convention should address the possibility that each lab may develop several WCBs and monoclonal antibody batches in-house. For this purpose, numbers can be added to the name.
- Institutions and individual Principal Investigators (PI) should be able to trace their WCB or monoclonal antibody back to the RM received from NIST.

## Proposed naming convention

Following the guidelines and assumptions discussed above, we suggest the following naming conventions:

## NISTCHO

Examples of the proposed NISTCHO nomenclature are shown in Table 1. If using NISTCHO propagated in a laboratory from RM 8675 NISTCHO acquired from NIST, it should be referred to as NISTCHO-XXXX-YYYY-ZZ, where

**Table 1.** Examples of the proposed NISTCHO nomenclature.

NISTCHO prefix	Company/ University	XXXX Identifier	Lab/department	YYYY Identifier	ZZ identifier	Naming Convention
NISTCHO	CloneCraft Biotech	CCB	John Smith	JS	2B	NISTCHO-CCB-JS-2B
NISTCHO	EchoBio	EB	Jane Doe	JD	1C	NISTCHO-EB-JD-1C
NISTCHO	NIST	NIST	Biomolecular Measurement Division	BMD	3D	NISTCHO-NIST-BMD-3D
NISTCHO	California College	CC	Department of Structural Biology	DSB	9B	NISTCHO-CC-DSB-9B
NISTCHO	NIIMBL	NIIMBL	John Doe	JD	B1	NISTCHO-NIIMBL-JD-B1

XXXX is at least a 2-digit alphanumeric company/university, or institute-specific identifier;
YYYY is at least a 2-digit alphanumeric identifier chosen by the lab, e.g., lab/department, or PI name; and
ZZ is a 2-digit alphanumeric identifier when more than one WCB is made by a lab/department or PI in the company, university, or institute.

## cNISTmAb

Examples of the proposed cNISTmAb nomenclature are shown in Table 2. When using the RM 8672 cNISTmAb acquired from NIST, it should be referred to as RM 8672 cNISTmAb.

**Table 2.** Examples of the proposed cNISTmAb nomenclature.

cNISTmAb prefix	Company/ University	XXXX Identifier	Lab/department	YYYY Identifier	ZZ	Naming Convention
cNISTmAb	Nexus Antibody	NA	John Smith	JS	1A	cNISTmAb-NA-JS-1A
cNISTmAb	PurePath Biologics	PPB	Jane Doe	JD	A3	cNISTmAb-PPB-JD-A3
cNISTmAb	California College	CC	Department of Cell Biology	DCB	C2	cNISTmAb-CC-DCB-C2
cNISTmAb	NIST	NIST	Biomolecular Measurement Division	BMD	1B	cNISTmAb-NIST-BMD-1B
cNISTmAb	NIIMBL	NIIMBL	John Doe	JD	D2	cNISTmAb-NIIMBL-JD-D2

When using cNISTmAb protein derived from a bioprocess using NISTCHO in a laboratory, it should be referred to as cNISTmAb-XXXX-YYYY-Z, where

XXXX is at least a 2-digit alphanumeric company, university, or institute-specific identifier;
YYYY is at least a 2-digit alphanumeric identifier chosen by the lab, e.g., lab/department, specific growth or media abbreviation, or PI name; and
ZZ is a 2-digit alphanumeric identifier of the lot made by a lab/department or PI in the company, university, or institute.

## Additional information on the cell lines and products

Published reports should include all relevant additional information on the NISTCHO and cNISTmAb used in the study in the Material and Methods section. Key information relating to NISTCHO typically includes the passage number (P), media, growth conditions (e.g., CO_2_, temperature), growth vehicle (e.g., shake flasks, bioreactor), and volume. cNISTmAb information should include the source of material (i.e., how the NISTCHO cells were grown and harvested), protein purification protocols, storage, and buffers.

## Concluding remarks

In contrast to other NIST reference materials, NISTCHO and cNISTmAb derivatives will be propagated and produced in different laboratories using different protocols, which will result in similar, but not identical, products. Therefore, a clear naming convention that is accepted and followed by the community will help identify the origin of particular cells or products and allow these materials to be traceable back to the NIST RMs. Use of a standardized naming convention will promote consistency in reporting and prevent confusion. We hope that this simple, straightforward convention will be adopted by the scientific community.

## Dedication

The authors wish to dedicate this paper to the memory of Franklin “Frank” Swartzwelder, who was a driving force behind the development of NISTCHO.
